# High prevalence of primary adrenal insufficiency in Zellweger spectrum disorders

**DOI:** 10.1186/s13023-014-0133-5

**Published:** 2014-09-02

**Authors:** Kevin Berendse, Marc Engelen, Gabor E Linthorst, AS Paul van Trotsenburg, Bwee Tien Poll–The

**Affiliations:** Department of Paediatric Neurology, Emma Children’s Hospital/Academic Medical Centre, Meibergdreef 9, Amsterdam, 1105 AZ The Netherlands; Laboratory for Genetic Metabolic Diseases, Emma Children’s Hospital/Academic Medical Centre, Meibergdreef 9, Amsterdam, 1105 AZ The Netherlands; Department of Endocrinology and Metabolism, Emma Children’s Hospital/Academic Medical Centre, Meibergdreef 9, Amsterdam, 1105 AZ The Netherlands; Department of Paediatric Endocrinology, Emma Children’s Hospital/Academic Medical Centre, Meibergdreef 9, Amsterdam, 1105 AZ The Netherlands

**Keywords:** Adrenal insufficiency, peroxisomal disorders, Very long-chain fatty acids, Zellweger spectrum disorders, X-linked adrenoleukodystrophy

## Abstract

Zellweger spectrum disorders are a group of autosomal recessive disorders characterized by impaired peroxisome functions. The clinical spectrum is broad, ranging from the classical most severe Zellweger syndrome to patients with a relatively mild phenotype. Treatment options are limited to symptomatic and supportive therapy. During routine follow-up we discovered patients with asymptomatic primary adrenal insufficiency. It is important to detect impaired adrenal function because it has treatment implications. Primary adrenal insufficiency was found in 7/24 patients examined, with 4/7 being asymptomatic. Systematic evaluation of adrenal function, through a Synacthen test, should be included in the clinical management of these patients.

**Electronic supplementary material:**

The online version of this article (doi:10.1186/s13023-014-0133-5) contains supplementary material, which is available to authorized users.

## Background

Peroxisomal disorders are a group of genetic disorders with impairment in one or more peroxisomal functions [1]. The peroxisomal disorders are divided into two major categories: (1) the peroxisome biogenesis disorders with multiple metabolic abnormalities (e.g. Zellweger spectrum disorders (ZSDs), OMIM #601539), and (2) the disorders with a deficiency of a single peroxisomal enzyme or transporter (e.g. X-linked adrenoleukodystrophy (X-ALD), OMIM #300100). X-ALD is characterized by accumulation of very long-chain fatty acids (VLCFA, ≥C22:0), due to an impaired peroxisomal beta-oxidation. Adrenal insufficiency, either subclinical or overt as well as the late onset form, is found in the majority of X-ALD patients, and frequently precede the neurological symptoms giving rise to the “Addison-only” phenotype [2].

Adrenal insufficiency may also occur in ZSD patients. The ZSDs are autosomal recessive disorders, characterized by impairment of multiple peroxisomal functions due to mutations in different *PEX* genes [1]. Clinically, the ZSDs constitute a spectrum of disease severity, ranging from severe (Zellweger syndrome (ZS), OMIM #214100) to attenuated phenotypes, like infantile Refsum disease (IRD, OMIM #266510) [3]. Most ZSD patients have disorders in the milder end of the spectrum and should be follow-up for symptomatic and supportive therapy. Among others, these patients have elevated concentrations of VLCFA and bile acid intermediates in plasma.

During routine follow-up we discovered impaired adrenocortical function in some of our ZSD patients. Because most of the patients have severe neurological symptoms, primary adrenal insufficiency might be easily overlooked. Untreated adrenocortical dysfunction may have serious consequences, while treatment is available [4]. To estimate the prevalence of adrenocortical dysfunction and, with that, to determine whether or not routine testing in these patients is useful, we assessed the adrenal function in our patients with a ZSD.

## Findings

The characteristics of the patients are presented in Table 1. Twenty-four patients with a ZSD, age ranging from 2.8-34.6 years (median 15.4 years) underwent a Synacthen test (Additional file 1). An inadequate increase in plasma cortisol concentrations was found in 7/24 (29%) patients and an adequate response in cortisol (>550 nmol/l) was seen in the others. Four of the 7 patients (57%) diagnosed with primary adrenal insufficiency, were asymptomatic. The 3 patients with clinical manifestations of adrenal insufficiency presented with muscle and/or joint pain, vomiting or hyperpigmentation. We found one patient (number 4) with additional adrenocortical dysfunction (i.e. hyperkalemia) as a sign of hypoaldosteronism [5]. Plasma renin was normal in 6/7 patients. Oral hydrocortisone (and fludrocortisone in patient 4) treatment was started in all diagnosed patients.

**Table 1:** **Clinical, laboratory and genetic findings in 24 patients with a mild ZSD**

	**Case**	**Present age,** ***y***	**Age at test,** ***y***	**C26:0 in plasma around time of Synacthen**	Average C26:0 in plasma*	**Basal ACTH**	**Basal cortisol /30’/60’ after injection**	**Clinical symptoms**	**Plasma renin levels**	**Phenotype**		***PEX*** **mutation** ^**1**^
										**Communication**	**Motor function**	
				***(0.45-1.32)***	***(0.45-1.32)*** **; STD**	***(1–55)***	***(>550)***		***(0–7.5)***			
**Adrenal insuf.**	1	6.6	5.9	**10.28**	**7.75**; 1.57	**100**	203 / 275 / **290**	asymp	5.5	++	++	*PEX1* c.2528G > A
	2	4.4	2.8	**7**	**6.28**; 1.13	**180**	201 / 254 / **260**	asymp	5.5	─	─	*PEX1* c.2528G > A
	3	20.4	18.5	**4.63**	**5.45**; 2.47	#	112 / 273 / **306**	asymp	5.1	─	─	*PEX1* c.2528G > A/ c.2097insT
	4	8.4	3.0	**4.43**	**4.93**; 0.99	#	200 / 260 / **190**	v	>576	─	+	*PEX1* c.2528G > A
	5	14.5	12.2	**3.38**	**3.87**; 1.37	**5140**	185 / 203 / **167**	hp	4.9	─	++	*PEX1* c.2528G > A
	6	24.7	22.9	**2.73**	**2.90**; 0.78	**66**	156 / 203 / **202**	hp, mp	2.7	++	─	*PEX26* c.292C > T
	7	11.8	10.1	**2.28**	**2.40**; 0.34	**390**	535 / 477 / **524**	asym	6.3	+	++	*PEX1* c.2528G > A
	8	24.3	24.2	**2.44**	**4.91**; 3.98	53	428 / 734 / 781			+	─	*PEX1* c.2528G > A
	9	24.2	23.8	**2.79**	**4.13**; 1.10	44	441 / 624 / 624			+	─	*PEX1* c.2528G > A/ unknown
	10	6.8	5.8	**3.38**	**3.94**; 1.06	nd	370 / nd / 990			─	─	*PEX1* c.2528G > A
	11	10.3	9.1	**3.78**	**3.94**; 1.15	17	125 / 458 / 607			─	─	*PEX1* c.2528G > A/ c.2097insT
	12	8.7	8.2	**3.21**	**3.77**; 0.69	35	85 / 425 / 557			++	++	*PEX1* c.2528G > A
	13	29.8	29.4	**3.7**	**3.71**; 0.78	27	902 / 1079 / 1090			++	+	*PEX1* c.2528G > A/ unknown
	14	22.8	22.2	**3.51**	**2.69**; 1.28	26	306 / 629 / 701			++	++	*PEX1* c.2528G > A
	15	17.3	16.9	**2.35**	**2.45**; 0.46	20	270 / 601 / 698			++	++	*PEX1* c.2528G > A
	16	35.0	34.6	**3.31**	**2.17**; 0.67	9	342 / 624 / 648			++	++	*PEX1* c.2528G > A
	17	19.0	18.6	**1.63**	**1.82**; 0.46	25	430 / 668 / 778			++	++	*PEX1* c.2528G > A
	18	8.1	7.5	1.24	**1.78**; 0.18	44	571 / 726 / 690			++	++	*PEX1* c.2528G > A
	19	11.8	11.2	1.2	**1.53**; 0.35	33	189 / 601 / 687			++	++	*PEX1* c.2528G > A
	20	8.3	7.6	0.81	**1.50**; 0.74	34	588 / 910 / 993			++	++	*PEX10* c. 1A > G/ c.199C > T
	21	29.7	29.3	**1.38**	**1.38**; **	nd	215 / 620 / 705			++	++	*PEX11*β c.64C > T
	22	15.4	14.6	**1.8**	**1.38**; 0.27	18	227 / 519 / 618			++	++	*PEX26* c.292C > T
	23	30.3	29.9	**1.98**	**1.35**; 0.39	19	274 / 579 / 709			++	++	*PEX1* c.2528G > A
	24	16.7	16.1	1.28	**1.34**; 0.34	17	356 / 593 / 665			++	++	*PEX1* c.2528G > A

Asym = asymptomatic; hp = hyper pigmentation; mp = muscle pain; nd = not determined; STD = standard deviation, v = vomiting. ACTH in ng/l, cortisol in nmol/l, C26:0 in μmol/l, renin in ugA1/L/U.

The reference intervals are indicated between brackets and all results outside the intervals are depicted in **bold.**

* = average concentration of C26:0 in plasma during life, ranging from 1 analysis to 43 (median 12) analyses per patient, ** = only one C26:0 measurement, # = unreliable measurement, i.e. loss of ACTH immunoreactivity caused by hemolysis. Phenotype: **-** = no communication or wheelchair bound, **+** = non-verbal communication or walk with support, **++** = verbal communication or independent walking. ^1^ = *PEX* mutations are homozygous if one sequence is given.

To obtain a framework for comparing severity of the clinical phenotype and adrenal insufficiency we delineated three categories on the basis of the degree of communication and motor function (Table 1). Elevated plasma VLCFA (C26:0) concentrations were present in 22/24, with an abnormal C26:0/C22:0 ratio in 24/24. Baseline plasma cortisol concentrations ranged from 85–902 nmol/l (median 272 nmol/l) and baseline plasma ACTH concentrations from 9–390 ng/l (median 33 ng/l), with one patient having 5140 ng/l.

**Figure 1:**
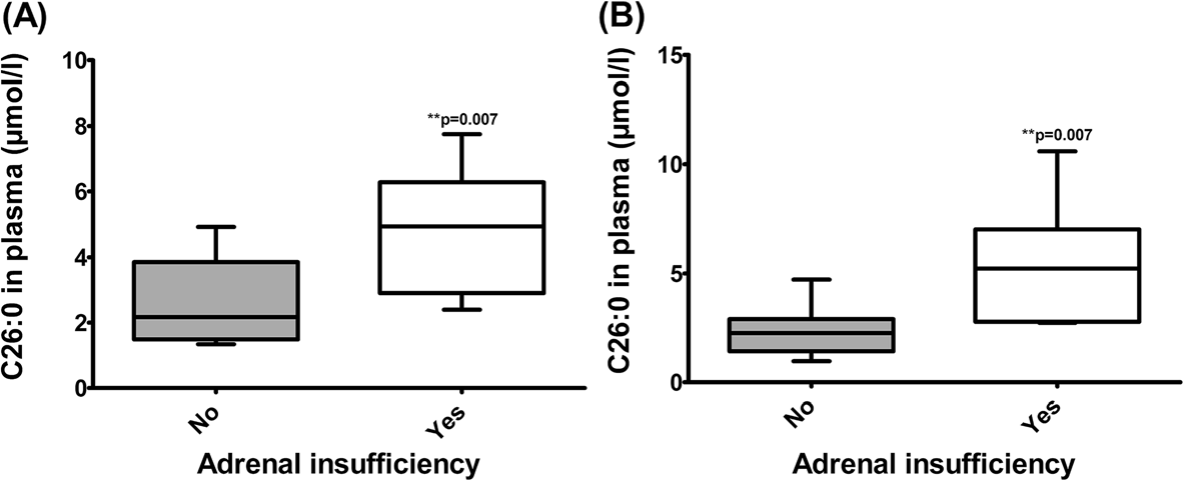
**Boxplots showing median, range and interquartile range of the VLCFA concentrations in plasma of the patients with or without a primary adrenal insufficiency. (A)** Mean of all VLCFA concentrations in time per patient **(B)** VLCFA concentrations around time of ACTH stimulation test. Statistical analyses were performed with a Mann–Whitney *U* test.

We found a correlation between the average concentrations of C26:0 and C26:0 levels around the time of the ACTH test in plasma and the occurrence of an adrenal insufficiency (Figure 1). There was no correlation between age, sex, *PEX* mutation or phenotypic severity and adrenal dysfunction.

## Discussion

In 1984, Govaerts et al. reported the occurrence of primary adrenal insufficiency in a small series of severe ZS patients [6]. The prevalence in ZSD patients with attenuated phenotypes has not yet been investigated. In this study we found a high prevalence of primary adrenal insufficiency in a cohort of ZSD patients.

The pathophysiological mechanism underlying the primary (usually only glucocorticoid) adrenocortical dysfunction in ZSDs and X-ALD is unknown. Several studies, including studies on post mortem material, suggest that elevated (i.e. toxic) concentrations of VLCFA cause adrenal cell dysfunction leading to decreased cortisol response and thus adrenal insufficiency [7-13]. We found a correlation between the occurrence of an adrenal insufficiency and the concentration of C26:0 in plasma. Because plasma C26:0 concentrations can fluctuate over time due to for instance diet, we used the average of all C26:0 values over time for each patient (Figure 1). We observed no adrenal insufficiency when the average concentration of C26:0 in plasma was below 2.40 μmol/l, while all patients with an average C26:0 > 4.91 μmol/l had adrenocortical dysfunction (Table 1, n = 4). Due to the single time point data, small size of our cohort and large variation in age, the importance of the correlation between VLCFA concentrations in plasma and adrenal insufficiency should not be overrated and needs confirmation in future studies.

In this study we found a high prevalence (29%) of primary adrenal dysfunction in ZSD patients, with some being asymptomatic at the time of the test. We therefore recommend that all ZSD patients should be screened for adrenal insufficiency during routine follow-up, in order to initiate appropriate therapy (Additional file 2).

## Additional files

Additional file 1:
**Supplementary methods.**
Additional file 2:
**Recommendations on the management of primary adrenal insufficiency.**

## Abbreviations

IRD: Infantile Refsum disease
VLCFA: Very long chain fatty acids
X-ALD: X-linked adrenoleukodystrophy
ZS: Zellweger syndrome
ZSD: Zellweger spectrum disorder
