# Supplementary material for: High prevalence of primary adrenal insufficiency in Zellweger spectrum disorders
Source: Orphanet J Rare Dis. 2014 Sep 2;9:133. doi: 10.1186/s13023-014-0133-5 (PMC4164755; doi:10.1186/s13023-014-0133-5)
Supplement: Additional file 1: — Supplementary methods. [file 13023_2014_133_MOESM1_ESM.pdf]

Additional file 1: Supplementary methods

All patients were followed in the outpatient clinic of the Academical Medical Centre (AMC), The Netherlands. Admission required biochemical and genetic proof of a ZSD. Having noticed several cases with adrenal dysfunction we included a total of thirty-six ZSD patients with no history of adrenal dysfunction at regular follow-up visits to guarantee complete medical care. Twelve patients had to be excluded because the test could not be performed (e.g. due to their severe cognitive impairment). Adrenocortical function was investigated by means of a “classical” ACTH stimulation (Synacthen) test [1], conducted according to the standard AMC protocol. During the ACTH stimulation test 250 µg Cosyntropin (1-24 ACTH) was injected intravenously and plasma cortisol concentrations were measured before, 30 and 60 minutes after injection (all blood samples were drawn between 9.00 AM and 2.30 PM). ACTH concentration was measured before injection. Primary adrenal insufficiency was considered to be present when the maximum cortisol concentration was lower than 550 nmol/l in the presence of a raised ACTH level. Cortisol and ACTH concentrations were determined in heparin and EDTA plasma, respectively, in the laboratory for endocrinology of the AMC. Cortisol (basal reference interval: 100-650 nmol/l) and ACTH (reference interval: 1-55 ng/l) concentrations were measured by in-house immunoassay methods.

VLCFA concentrations in plasma were determined as previously described by Dacremont et al [2]. The average C26:0 concentration in plasma was calculated using a total of 1 to 43 (median 12) biochemical analyses per patient. Complementation analysis followed by Sanger sequencing was done for each patient on genomic DNA and the mutation was confirmed in parents/family in 20/24 patients. Reference sequence of PEX1, PEX10 and PEX26 are respectively NM_000466.2, NM_002617.3 and NM_017929.4. Nucleotide numbering starting at the first adenine of the translation initiation codon ATG. For statistical analysis, Mann-Whitney U tests were performed using the IBM Statistical package for the Social Sciences (SPSS) software version 20 (IBM, U.S.A.).

1. Eik-nes BYK, Sandberg AA, Nelson DONH, Frank H, Samuels LEOT: **Changes in plasma levels of 17-hydroxycorticosteroids during intravenous adminitration of ACTH. I. A test of adrenocortical capacity in the human**. *J Clin Invest* 1954, **33**:1502–1508.

2. Dacremont G, Cocquyt G, Vincent G: **Measurement of very long-chain fatty acids, phytanic and pristanic acid in plasma and cultured fibroblasts by gas chromatography.** *J Inherit Metab Dis* 1995, **18 Suppl 1**:76–83.
