# Supplementary material for: High prevalence of primary adrenal insufficiency in Zellweger spectrum disorders
Source: Orphanet J Rare Dis. 2014 Sep 2;9:133. doi: 10.1186/s13023-014-0133-5 (PMC4164755; doi:10.1186/s13023-014-0133-5)
Supplement: Additional file 2: — Recommendations on the management of primary adrenal insufficiency. [file 13023_2014_133_MOESM2_ESM.pdf]

Additional file 2: Recommendations on the management of primary adrenal insufficiency

The treatment of adrenal insufficiency in the Academic Medical Centre, at the department of Paediatric Endocrinology, consists of oral administration of the glucocorticoid hydrocortisone (cortisol) in a dose of approximately 10 mg per m^2 per day, and (often) the mineralocorticoid fludrocortisone (50 to 200 microgram per day in one or two doses). Fifty percent of the total daily hydrocortisone dose should be administered in the morning, 25 percent between noon and 2:00 PM, and 25 percent between 4:00 to 8:00 PM. Extra doses should be given under time of physical stress, including infection, trauma or anesthesia. Threefold the doses when stress is moderate, fivefold when severe (e.g. high fever). Furthermore, the total daily dose should then be equally divided in four six-hourly administrations. In case of an adrenal crisis, i.e. severe signs and symptoms of cortisol deficiency like, persistent vomiting, breathing fast, hypotension and even loss of consciousness, hydrocortisone should be administered parenterally (intramuscular or intravenous) without delay. In infants the first dose should be 25 mg, between the ages of 1 and 6 years 50 mg, and thereafter 100 mg. Because of its life threatening character, adrenal insufficiency is an indication for wearing a Medical Alert bracelet at all times that indicates that he/she has an adrenal insufficiency. To exclude mineralocorticoid deficiency, plasma aldosterone and renin should be measured and monitored.
